# The Urgent Need for Clinical Nutrition Education in Medical Training: Integrating Developmental Origin of Health and Disease and Perinatal Nutrition into Programs and Credentialing

**DOI:** 10.1016/j.advnut.2025.100450

**Published:** 2025-05-23

**Authors:** Ronilson Corrêa, Ana Elisa Toscano, Paula Brielle Pontes, Raul Manhães de Castro

**Affiliations:** 1 Postgraduate degree in Mental Health from Dom Bosco Catholic University, Campo Grande, Brazil; 2 Postgraduate Program in Neuropsychiatry and Behavior Science, Federal University of Pernambuco—UFPE-Recife, Pernambuco, Brazil; 3 Studies in Nutrition and Phenotypic Plasticity Unit, Federal University of Pernambuco, Recife, Pernambuco, Brazil; 4 Department of Nursing, Federal University of Pernambuco—UFPE, Vitória of Santo Antão, Pernambuco, Brazil; 5 Department of Nutrition, Center for Health Sciences, Federal University of Pernambuco, Recife-Pernambuco, Brazil

Dear Editor:

We developed a profound interest in the subject upon reading the article “Addressing the Urgent Need for Clinical Nutrition Education in Postgraduate Medical Training” by Krishnan et al. This article underscores the pressing global crisis, particularly in the United States, regarding the nutritional training of physicians. The authors’ innovative solutions, such as the Duke Online Clinical Nutrition Fellowship, are commendable. We appreciate their pragmatic approach and their advocacy for interdisciplinary curricula that place a greater emphasis on clinical nutrition. We strongly believe that interdisciplinary collaboration is key to addressing this urgent issue.

However, I would like to broaden the discussion and suggest the inclusion of the Developmental Origin of Health and Disease theory. This theory, in simple terms, suggests that the conditions a fetus experiences in the womb can have a significant impact on its health later in life. A study by Barker [1] demonstrated that nutritional deficiencies (e.g., fatty acids, iron, and vitamin D) alter fetal brain development [2]. However, doctors are rarely trained to interpret these mechanisms or prescribe dietary interventions.

Developmental research on the origin of health and disease (Developmental Origin of Health and Disease) demonstrates that experiences such as maternal stress during pregnancy can program the child’s brain development, influencing areas such as the hippocampus, which affects cognitive and socioemotional skills [2]. Phenotypic neuroplasticity is the ability of an organism to adjust its development and characteristics in response to the environment, as occurs in the fetal brain in response to maternal stress during pregnancy [2]. In the perinatal phase, the brain is most vulnerable to environmental factors and nutritional deficiencies [1,2]. Factors such as protein–calorie malnutrition; nutrients such as essential fatty acids, iron, retinol (vitamin A), and vitamin D [2]; and environmental insults such as perinatal distress and stress [3,4] can impair the development of fetal brain growth [5], metabolism, and cortical maturation leading to lifelong health consequences such as metabolic diseases, cardiovascular diseases [5,6], and neuropsychiatric disorders, such as schizophrenia [7].

Maternal malnutrition reprograms the hypothalamic–pituitary–adrenal axis of the fetus, increasing the risk of anxiety and eating disorders. The maternal diet influences the epigenetic programming of the fetus [4]. Adequate nutrition during the perinatal phase is essential for neurologic development [1,3]. Phenotypic neuroplasticity makes the brain more susceptible to interventions during critical periods of development, such as childhood and adolescence [2]. In view of this evidence, nutritional care programs in the perinatal phase can be used and adopted to ensure adequate nutrition during this period, mitigating many of these negative effects.

In this sense, perinatal nutrition is a primary prevention, as phenotypic neuroplasticity makes the perinatal period a window of opportunity for prevention [1]. Community-based programs that combine nutritional counseling reduce cases of postpartum depression by ≤30% [8]. However, such strategies require physicians to understand the nutrition–brain interface, which requires the insertion of mandatory nutrition modules in medical courses and postgraduate courses in psychiatry and obstetrics.

Medical training cannot ignore the knowledge of nutrition in cases of hypertension and the prevention of strokes. Doctors need to be trained to prescribe folic acid and nutritional supplementation such as iron to anemic pregnant women who are aware of its impact on infant cognition.

We congratulate the authors for their significant study and for reinforcing the importance of including nutritional aspects in health strategies from the perinatal phase for prevention and lifelong treatment. We believe that future implementations of the model, with the integration of clinical nutrition into undergraduate and graduate curricula, can empower medical professionals from screening to treatment. The practical application of this knowledge can significantly improve the understanding and management of health from the perinatal phase, giving us hope for a healthier future. We would be honored if the authors could respond to this letter and engage in productive discussions with us.

## Author contributions

The authors’ responsibilities were as follows – RC: idealized the study; RC, PBP: wrote the manuscript; AET, RMdC: edited and supervised the study; and all authors: have read, revised, and approved the final manuscript.

## Declaration of generative AI and AI-assisted technologies in the writing process

During the preparation of this work, the author(s) used Grammarly in order for translation. After using this tool/service, the author(s) reviewed and edited the content as needed and take(s) full responsibility for the content of the publication.

## Funding

Supported in part by the Foundation for the Support of Science and Technology of Pernambuco (FACEPE, 0989-4.05/22 and 1471-4.05/22), Brazil; the National Council for Scientific and Technological Development (CNPq; 402426/2021-5 and 404181/2023-6), Brazil; and the Coordination for the Improvement of Higher Education Personnel (CAPES; Finance Code 001 and 1238/2022/88881.707895/2022-01), Brazil.

## Conflict of interest

The authors declare no conflicts of interests.
